# Correlation between the accumulation of skin glycosylation end products and the development of type 2 diabetic peripheral neuropathy

**DOI:** 10.1186/s12902-022-00997-6

**Published:** 2022-04-20

**Authors:** Xing-Wang Zhao, Wan-Xu Yue, Sen-Wei Zhang, Qiu Chen

**Affiliations:** 1 Jianyang Hospital of Traditional Chinese Medicine, Jianyang, 641400 China; 2 Hospital of Chengdu University of Traditional Chinese Medicine, Chengdu, 610072 China; 3 Chengdu University of TCM, Chengdu, 611137 China

**Keywords:** Advanced glycation end products, Detection method, Illness assessment

## Abstract

**Background:**

The accumulation of advanced glycation end products (AGEs) occurring in skin tissues can be measured by AGE Reader. Here, we assessed the correlation between AGEs values and the development of type 2 diabetic peripheral neuropathy (DPN).

**Methods:**

The basic clinical information of 560 patients with T2DM was collected through an electronic system. AGEs and diabetic complication risk score was measured by AGE Reader, a non-invasive optical signal detector. All of the participants were classified into 4 groups based on Dyck criteria: grade 0 (non-DPN group), grade 1 (early stage group), grade 2 (middle stage group) and grade 3 (advanced group). Pearson correlation analysis and Spearman correlation analysis were used to evaluate the correlation between AGEs and other indexes. The sensitivity and specificity of glycosylated products were evaluated by ROC curve.

**Results:**

With the increase of DPN severity, the accumulative AGEs showed an increasing trend. Significant differences (*P* = 0.000) of AGEs were found among grades 0, 1, 2, and 3 of DPN, and significant differences (*P* = 0.000) of AGEs were found between grades 1 and 3. There were significant differences in DPN risk score between grades 0, 1, 2, and 3, between grades 1, 2, and 3, and between grades 2 and 3 (*P* < 0.01 or *P* < 0.05). AGEs were positively correlated with age, blood uric acid, disease course, systolic blood pressure, the risk scores of the four major complications of diabetes, renal function indicators (serum creatinine, Cystatin C, homocysteine, the ratio of urinary albumin and creatinine, urinary microalbumin, α-microglobulin, urinary transferrin, urinary immunoglobulin), inflammatory indicators (white blood cell count, neutrophil count, neutrophil-to-lymphocyte ratio, C-reactive protein), and TCSS score. However, it was negatively correlated with BMI,fasting insulin, insulin 1–3 h postprandial, lymphocyte count, HOMA insulin resistance index and estimated glomerular filtration rate. The area under the AGEs cumulant and neuropathy risk score curve was 0.769 and 0.743, respectively. The confidence intervals were (71.2–82.6%) and (68.8–79.9%), respectively. The maximum Youden’s index of AGEs cumulant was 0.440, and the corresponding AGEs cumulant value was 77.65. The corresponding sensitivity and specificity were 0.731 and 0.709, respectively. Furthermore, the maximum Youden’s index of neuropathy risk score was 0.385, and the corresponding neuropathy risk score was 66.25. The corresponding sensitivity and the specificity were 0.676 and 0.709, respectively.

**Conclusion:**

The cumulative amount of skin AGEs can be used as the diagnostic index and the prediction and evaluation index of DPN severity. Moreover, the diabetic peripheral neuropathy risk score can predict the risk of DPN in patients with T2DM.

## Introduction

Diabetic peripheral neuropathy (DPN) is a common chronic complication of diabetes, and its incidence rate is also increasing, which is an essential cause of foot ulcer, infection, arterial thrombosis, muscular atrophy, fall fracture, gangrene and even amputation. The ineffective or poor treatment of DPN increases the expenditure of patients with diabetes and also causes the waste of national medical resources. Thus, conducting diabetes screening as early as possible and evaluating the risk value and severity of diabetes and its complications are of great significant.

AGEs plays a vital role in the evolution of diabetes and its complications. AGEs are covalent adders with high activity and stability produced by nonenzymatic glycation reactions (also known as Maillard reactions) without the involvement of enzymes. AGEs are highly heterogeneous, with several existing forms in human tissues and organs. Currently recognized structural forms include pentosidine, carboxymethyl lysine, carboxyethyl lysine pyrraline and crossline.

Presently, the pathogenesis of diabetic peripheral neuropathy is not very clear. Clinical and animal data suggest that AGEs induced by hyperglycemia plays an important role in nerve injury [1], which is closely related to other metabolic mechanisms, including increasing its number through the polyol pathway, enhancing oxidative stress [2], and activating the diacylglycerol kinase C (PKC) pathway [3, 4]. All of these mechanisms contribute to increased oxidative stress, which increases the formation of glycation products, creating a vicious cycle. Other studies [5] have found that diabetic peripheral neuropathy, especially small-fiber neuropathy, is significantly correlated with the increase of AGEs in the skin. Dipino [6] et al. confirmed that a large part of the human body’s AGEs is exogenous, which is an essential source of the accumulation of AGEs in the body. Glycation toxicity caused by overcooking animal-derived foods has been reported under different pathological conditions, including chronic kidney disease and aging. Negrean et al. [7] demonstrated in a systematic analysis of type 2 diabetes patients that microvascular function was impaired after high-dose diets, and serum AGEs and oxidative stress markers were elevated.

The detection of AGEs in skin biopsies has been used to predict diabetes mellitus and its complications in a prior study [8] to realize the risk and disease assessment of diabetes complications. However, the invasive and time-intensive nature of skin biopsies has limited the application of this method for at-risk outpatients. Skin AGEs were assessed by an AGE Reader (Hefei Institutes of Physical Science, Chinese Academy of Sciences) following a 2–3-min measurement of the left volar forearm. AGE Reader uses the principle of positive correlation between the fluorescence intensity of specific excitation/emission band and AGEs accumulation amount in skin tissues to invert the AGEs level of subjects in vivo based on the obtained optical signals.

The dermis of the skin is rich in collagen. 370 nm excitation light can penetrate the epidermis to reach the superficial dermis and stimulate the collagen cross-links in the dermis to produce fluorescence. Because the excitation peak of collagen is at 370 nm and the emission peak is at 440 nm, the best wavelength to excite the fluorescence of AGEs in the skin is at 370 nm. In short, an excitation light source with a peak wavelength of 370 nm was used to excite the AGEs in the skin that have fluorescence properties in a frequency range of 420–600 nm. In order to reduce the influence of scattering and absorption on tissue fluorescence spectra, after tissue fluorescence and diffuse reflectance in different tissue optical properties were simulated by the Monte Carlo method, a tissue intrinsic fluorescence recovering algorithm using diffuse reflectance spectrum was developed. The empirical parameters in the tissue intrinsic fluorescence recovering algorithm were coded as a particle in the solution domain, the classification performance defined as the fitness, and then a particle swarm optimization (PSO) algorithm established for empirical parameters optimization. In addition, a support vector machine (SVM) method was implemented to improve the performance of the classification [9, 10].

Although the correlation between serum AGEs and neuropathy has been confirmed, only a few small sample case studies [11, 12] have shown a certain correlation between AGEs measured by skin fluorescence and type 1 diabetic peripheral neuropathy, which is mainly used in Caucasians. In Asian populations, the association between cumulative skin AGEs and DPN of varying severity in type 2 diabetic patients has not been reported in the literature.

### Experimental Procedures

#### Patients with type 2 diabetes

This study included 560 type 2 diabetes patients hospitalized in the department of endocrinology, hospital of Chengdu University of Traditional Chinese Medicine from May 2016 to June 2018. The participants included 312 and 248 men and women, respectively. All experiments on human subjects were conducted according to the Declaration of Helsinki and that all procedures were carried out with the subjects’ adequate understanding and written consent.

#### Inclusion and exclusion criteria

The inclusion criteria included the following: patients who meet the diagnostic criteria of T2DM in the WHO criteria for diabetes diagnosis and classification (1999), conform to the diagnostic criteria of DSPN published by ADA in 2010, aged > 18 years, and participate voluntarily.

Moreover, the exclusion criteria included the following:

1. Recent acute complications of DM, including DKA, severe hypoglycemia coma and co-infected patients;
2. Patients with severe liver function impairment (AST or ALT 2.5 times higher than the upper limit of normal), including patients with cirrhosis, severe hepatitis or severe cardiovascular and blood diseases;
3. Neuropathy caused by other factors was diagnosed, including severe macrovascular abnormalities (thrombosis, etc.), cervical and lumbar problems (pinched nerve roots, narrow spinal canal, senile vertebral changes), cerebral infarction, Guillain-Barre syndrome, and neurotoxic and side effects caused by certain drugs. The accumulation of toxic substances in the body caused by renal insufficiency causes nerve damage;
4. Patients with other endocrine diseases that can significantly increase blood glucose, including hyperthyroidism, cortisolism, Cushing’s syndrome, or those who have recently used glucocorticoids;
5. Patients with mental illness, deformity or other reasons that cannot complete the test with the assistance of the detection personnel;
6. Patients with a large area of scar, rash, vitiligo, or other infectious skin diseases on the ulnar skin of the left forearm.

Samples will be eliminated from the study if patients do not meet the corresponding criteria or the data is incomplete.

### Research method

#### Detection methods of skin fluorescence AGEs

Test process: after calibrating the instrument, patients must fill in their basic information. Subjects will be seated at a constant room temperature (approximately 25 °C) with their left forearm flat on the arm limit bracket, palms down, and the inner forearm skin (approximately 10–15 cm below the elbow) attached to the scanner probe. The forearm should be raised with the elbow as the fulcrum between each of the three scans until the forearm is removed from the probe and the forearm position is restored. The instrument automatically averages the three results and estimates the risk of four major complications. Note: The skin should not have visible blood vessels, scars, moss-like plaques, vitiligo, malformation, or other skin abnormalities. If the arm skin has thick hair, it needs to be tested after shaving. Do not apply care cream or any fluorescent substance, including sunscreen or body lotion, and wash and dry with clean water before testing.

#### Grouping criteria

Participants were grouped according to the P. J. Dyck criteria, full box results, and the Toronto Clinical Scoring System (TCSS).

P. J. Dyck criteria described the severity stages of neuropathy as follows: grade 0 (non-DPN group), diabetic group without neuropathy; grade 1 (early stage), asymptomatic neuropathy; grade 2 (middle stage), neuropathic symptoms are present, but without neurological involvement; and grade 3 (advanced), presence of neuropathic symptoms and functional involvement [13].

For full box results, vibration perception threshold (VPT) of < 15 is the low-risk group, 15–25 is the medium-risk group, and > 25 is the high-risk group. According to the current perception threshold (CPT), it was divided into normal group, attenuated group and sensitive group.

For the TCSS, total scores of 0–5, 6–8, 9–11,and 12–19 points are classified as normal, mild, moderate, and severe, respectively [14].

#### Observational index

The general indicators for these patients included age, sex, course of illness, blood pressure, body mass index (BMI), HbA1c, fasting blood glucose (FBG), 1-h postprandial blood glucose (1-h PBG), 2-h postprandial blood glucose (2-h PBG), fasting C-peptide, 1-h standardized postprandial C-peptide (1-h C-peptide), 2-h standardized postprandial C-peptide (2-h C-peptide), fasting insulin, 1-h standardized postprandial insulin (1-h insulin) and 2-h standardized postprandial insulin (2-h insulin). In addition, Serum calcium (Ca^2+^), urea nitrogen (UA), serum creatinine (Cr), Cystatin C (CysC), homocysteine (Hcy), white blood cell count (WBC), neutrophil count (NEUT), lymphocyte count (LY), neutrophil-to-lymphocyte ratio (NLR), C-reactive protein (CRP), the ratio of urinary albumin and creatinine (ACR), urinary microalbumin (MAU), α-microglobulin(α-MG), urinary transferrin (Tf), urinary immunoglobulin (Ig), total cholesterol (TC), triglycerides (TG), high-density lipoprotein cholesterol (HDLC), low-density lipoprotein cholesterol (LDL-C), score of vibration perception threshold (VPT), score of current perception threshold (CPT), and Toronto clinical scoring system (TCSS) were detected. HOMA insulin resistance index (HOMA-IR) was calculated as [Plasma glucose (GLU, mmol/L)* serum insulin (mIU/L)] / 22.5. The Cockcroft-Gault equation was used to determine the estimated glomerular filtration rate (eGFR).

#### Data statistics and analysis methods

Continuous variables that conformed to a normal distribution are expressed as the mean ± standard deviation, while non-normally distributed variables are expressed as the median with the interquartile range. ANOVA and the Kruskal-Wallis tests were used for comparisons among groups. Pearson correlation analysis and Spearman correlation analysis were used to evaluate the correlation between AGEs and other indexes. The sensitivity and specificity of glycosylated products were evaluated by ROC curve. A two-tailed *P*-value of less than 0.05 was defined as statistically significant. All of the statistical analyses were performed using SPSS 20.0 statistical software. Statistical charts were made by GraphPad Prism and SPSS 20.0 statistical software.

## Research results

### Correlation of indicators among DPN groups

#### Normal data analysis

##### Results

One-way analysis of variance (ANOVA) was conducted for the data according to the normal distribution: the significance of age, AGEs, postprandial 1, 2 h insulin, and course of disease were all < 0.05, indicating that the above indexes had significant statistical differences in different groups (Table 1).

**Table 1:** Comparison of clinical and laboratory indicators between groups($\overline{\mathrm{X}}$ ±S)

Indicators	Grade 0 (***n*** = 79)	Grade 1 (***n*** = 199)	Grade 2 (***n*** = 202)	Grade 3 (***n*** = 80)	F	***P***
AGEs (AU)	74.36 ± 11.97	84.02 ± 13.88	87.72 ± 15.67	93.99 ± 19.83	24.290	.000*
Course (years)	2.96 ± 2.91	7.54 ± 6.48	10.27 ± 7.23	8.36 ± 5.30	4.032	.009*
Age (years)	50.2 ± 12.71	59.31 ± 11.26	62.06 ± 11.89	69.85 ± 10.87	39.423	.000*
Weight (kg)	62.91 ± 12.95	65.22 ± 11.51	63.58 ± 11.10	62.71 ± 10.31	1.420	.236
BMI (kg/m2)	24.31 ± 3.50	24.48 ± 3.34	24.42 ± 3.79	23.70 ± 3.12	1.031	.378
Ca^2+^(mmol/L)	2.21 ± 0.14	2.21 ± 0.13	2.19 ± 0.15	2.18 ± 0.13	1.293	.276
UA (μmol/L)	316.98 ± 100.82	336.52 ± 100.30	332.97 ± 100.92	355.74 ± 90.62	2.074	.103
TC (mmol/L)	4.62 ± 1.17	4.40 ± 1.16	4.40 ± 1.08	4.39 ± 1.36	.815	.486
LDL (mmol/L)	2.69 ± 0.91	2.57 ± 0.81	2.54 ± 0.78	2.54 ± 0.88	.617	.604
WBC (*10^9^/L)	6.05 ± 1.83	6.10 ± 1.93	6.14 ± 1.89	6.02 ± 2.17	.067	.978
1hPBG (mmol/L)	14.99 ± 4.24	14.27 ± 3.56	15.31 ± 2.72	15.02 ± 4.48	.506	.679
2hPBG (mmol/L)	17.71 ± 5.17	18.07 ± 4.25	17.29 ± 4.83	20.85 ± 6.18	2.453	.064
3hPBG (mmol/L)	15.97 ± 6.60	16.30 ± 4.62	18.52 ± 3.13	19.15 ± 4.95	1.716	.171
INS60’(mIU/L)	47.41 ± 54.60	25.75 ± 17.67	17.95 ± 10.10	13.08 ± 5.48	4.106	.009*
INS120’(mIU/L)	62.25 ± 70.87	28.90 ± 17.50	24.61 ± 14.71	15.93 ± 9.89	4.613	.005*
INS180’(mIU/L)	48.10 ± 54.17	28.66 ± 18.60	25.82 ± 15.55	19.35 ± 9.55	2.291	.085

* The significance level of the mean difference is 0.05

**Fig. 1:**
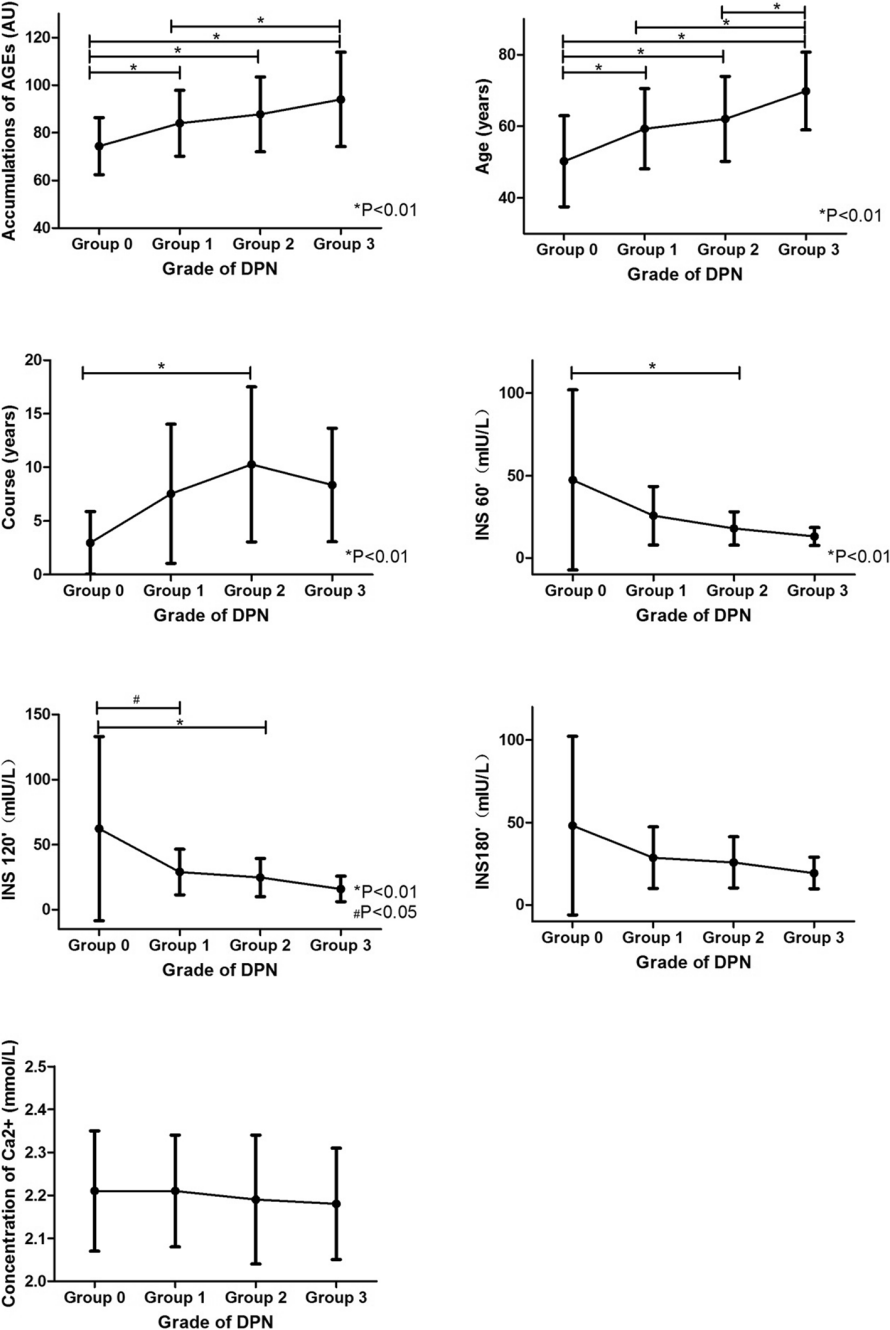
Relationship of general indicators among groups of DPN

Results (Fig. 1): ① Comparison of AGEs between groups: significant differences (*P* = 0.000) of AGEs were found among grades 0, 1, 2, and 3 of DPN, and significant differences (*P* = 0.000) of AGEs were found between grades 1 and 3. ② Comparison of general data between groups: significant age differences were found among grades 0,1, 2, and 3 (all *P* = 0.000), and between grades 1 and 3, 2 and 3 (all *P* = 0.000). The course of DPN grades 0 and 2 was significantly different (*P* = 0.007). Significant differences were also found between DPN grades 0 and grade 2 at 1 h postprandial insulin (P = 0.007), between grades 0 and 1, and between grades 0 and 2 at 2 h postprandial insulin (*P* = 0.012, *P* = 0.005). ③With the increase of DPN severity, the accumulative AGEs, age, and course of diseases all showed an increasing trend. Insulin decreased after 1 and 2 h postprandial. Additionally, although no significant statistical difference was found in calcium ion concentration and insulin level at 3 h postprandial, both showed a gradually declining trend as seen from the statistical figures.

#### Non-normal measurement data analysis

Results (Table 2): (1). Kruskal–Wallis H rank and inspection: TG, eGFR, Cr, CysC, Hcy, ACR, MAU, α-MG, Tf, U-Ig G, LY, NLR, and CRP showed significant differences among groups (*P* < 0.05), indicating that statistically significant differences between the two groups were found in the indicators mentioned above at least.

**Table 2:** Comparison of clinical and laboratory indicators between groups (median (lower quartile ~ upper quartile))

Variable	Grade 0 (***n*** = 79)	Grade 1 (***n*** = 199)	Grade 2 (***n*** = 202)	Grade 3 (***n*** = 80)	***P***
TG (mmol/L)	1.86 (1.13–2.93)^c#^	1.74 (1.13–2.71)^e#^	1.57 (1.07–2.40)	1.385 (0.92–2.12)	0.016*
HDL-C (mmol/L)	0.99 (0.82–1.16)	1.015 (0.84–1.16)	1.025 (0.87–1.22)	1.07 (0.93–1.32)	0.069
Cr (μmol/L)	59.1 (48.90–68.20)	63.25 (54.18–76.98)^e#^	61.95 (51.40–76.28)^f##^	73.5 (59.53–88.00)^c##^	0.000*
CysC (mg/L)	0.78 (0.67–0.89)^a##^	0.935 (0.77–1.21)^e##^	0.935 (0.80–1.18)^bf##^	1.09 (0.90–1.47)^c##^	0.000*
Hcy (μmol/L)	10.03 (7.21–12.47)^c##^	9.91 (7.81–13.26)	9.77 (8.22–12.88)^f##^	12.1 (9.55–15.79)^e##^	0.001*
ACR (mg/g)	12.35 (5.24–33.89)^c##^	14.46 (5.83–73.43)^e#^	16.63 (5.62–109.55)^f#^	51.21 (16.64–296.16)	0.005*
eGFR (ml/min)	109.59 (98.39–120.75)	97.9 (84.92–110.34)^ae##^	96 (80.98–108.94)^bf##^	84.84 (64.43–100.41)^c##^	0.000*
MAU (mg/L)	10.6 (5.34–25.60)^c##^	11.8 (5.15–62.00)^e##^	12.65 (4.36–51.03)^f##^	47.1 (9.39–239.50)	0.001*
α-MG (mg/L)	9.96 (55.25–15.73)^c##^	12.6 (5.30–25.80)	10.5 (5.01–24.10)^f#^	19.1 (7.79–34.30)	0.004*
Tf (mg/L)	2 (2.00–2.00)^c##^	2 (2.00–4.46)^e#^	2 (2.00–2.92)^f##^	2.12 (2.00–14.00)	0.000*
U-Ig G (mg/L)	3.75 (3.00–10.19)^c##^	5.36 (3.00–13.75)^e#^	4.36 (3.00–11.40)^f##^	9.15 (4.03–36.10)	0.001*
NEUT(10^9^/L)	3.305 (2.70–4.08)	3.5 (2.70–4.34)	3.62 (2.67–4.52)	3.74 (2.80–4.60)	0.468
LY(10^9^/L)	1.935 (1.45–2.54)	1.66 (1.25–2.17)^a#^	1.69 (1.25–2.04)^b#^	1.51 (1.26–1.84)^c##^	0.001*
NLR	1.64 (1.40–2.24)	2.01 (1.56–2.72)^a#^	2.13 (1.56–2.86)^b##^	2.415 (1.69–3.20)^c##^	0.000*
CRP (mg/L)	0.7 (0.50–1.80)	0.8 (0.50–2.58)	0.9 (0.50–2.60)	1 (0.60–7.60)^c#^	0.033*
HOMA-IR	1.86 (1.37–6.40)	1.82 (1.18–3.43)	2.005 (1.28–3.07)	0.93 (0.52–1.42)	0.257
PFG (mmol/L)	8.47 (6.38–11.35)	8.3 (6.41–10.17)	7.94 (5.94–10.53)	8.425 (6.25–11.53)	0.394
FINS (mIU/L)	6.78 (3.85–14.75)	7.005 (3.36–10.260	5.84 (3.93–8.59)	3.57 (2.29–4.46)	0.302
C-Peptide (nmol/L)	0.666 (0.45–0.83)	0.7275 (0.48–0.96)	0.646 (0.50–0.87)	0.554 (0.42–0.69)	0.158
2hC-peptide (nmol/L)	1.6 (1.22–2.47)	1.85 (1.29–2.53)	1.59 (1.10–2.10)	1.3 (0.79–2.06)	0.142
HbA1c(%)	9 (7.08–10.48)	8.35 (6.63–11.08)	7.8 (6.70–10.30)	7.35 (6.10–9.88)	0.103

^*^ The progressive significance level was 0.05. Significance of pared comparison after adjustment #*P* < 0.05; significance of paired comparison after adjustment ##*P* < 0.01. ^a ^comparison of grades 0 and 1; ^b ^comparison between grades 0 and 2; ^c ^comparison of grades 0 and 3; ^d ^comparison between levels 1 and 2; ^e ^comparison between levels 1 and 3; ^f ^comparison of levels 2 and 3

**Fig. 2:**
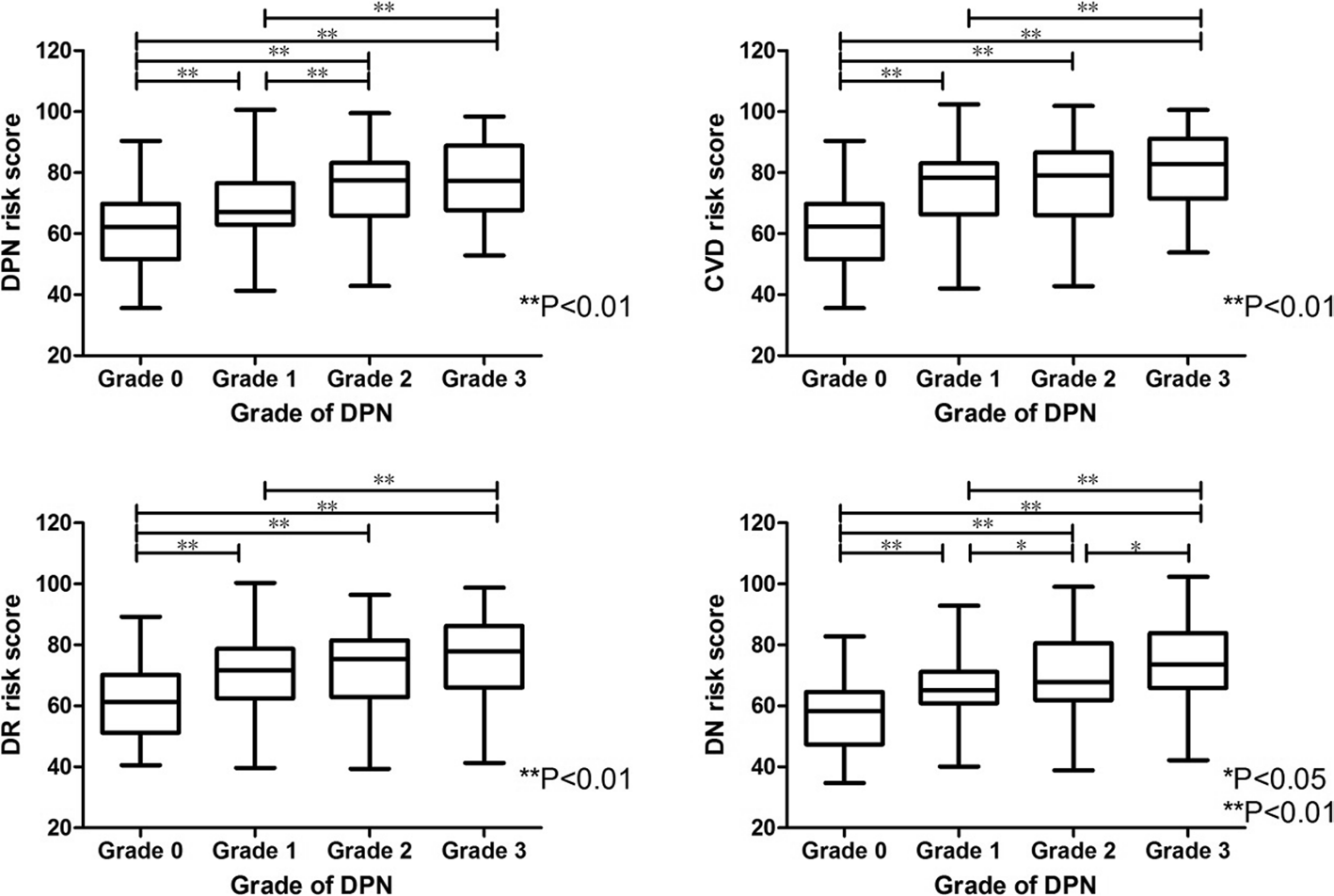
Correlation between diabetic complication risk score and diabetic peripheral neuropathy

(2) Pairwise comparison after adjustment: ①For lipid indicators, TG levels were significantly different between grades 3, 0, and 1 (*P* < 0.01 or *P* < 0.05). ②For renal function indicators, significant differences in eGFR were found between grades 0 1, 2, and 3 and between grades 3,1,and 2 (*P* < 0.01 or *P* < 0.05). Significant differences in Cr, ACR, Hcy, MAU, Tf, and Ig, were found between grades 0, 1, 2 and 3 (*P* < 0.01 or *P* < 0.05). Significant differences in CysC were found between grades 0, 1, 2, and 3, and between grades 1, 2 and 3 (*P* < 0.01). The difference of α-MG between grades 0, 2, and 3 was found to be significant (*P* < 0.01 or *P* < 0.05). ③For inflammation-related indicators, a significant difference in CRP was found between grades 0 and 3 (*P* < 0.05). Significant differences in LY and NLR were found between grades 0, 1, 2 and grade 3 (*P* < 0.01 or *P* < 0.05).

(Figure 2) Four risk scores of diabetes complications in different DPN groups: There were significant differences in DPN risk score between grades 0, 1, 2 and 3, between grades 1, 2 and 3, and between grades 2 and 3 (*P* < 0.01 or *P* < 0.05). Significant differences in CVD and DR risk score were found between grades 0, 1, 2 and 3, and between grades 1 and 3 (*P* < 0.01). Significant differences in DN risk scores were found between grades 0, 1, 2 and 3,between grades 1,2 and 3 (*P* < 0.01).

#### Correlation study of AGEs and other indicators

Pearson correlation analysis of the cumulative volume of skin AGEs showed that AGEs were positively correlated with age, blood uric acid, white blood cells, disease course and systolic blood pressure. However, it was negatively correlated with BMI and insulin 1–3 h postprandial (Table 3).

**Table 3:** Correlation analysis of AGEs and other indicators by Pearson

Indicators	r	***P***
Age	. 434**	.000
BMI	−.163**	.000
Ca^2+^	−.001	.984
UA	.175**	.000
TC	.003	.937
LDL	.021	.614
WBC	.139**	.002
1hPBG	.045	.696
2hPBG	.079	.217
3hPBG	.164	.150
INS60’	−.310**	.006
INS120’	−.302**	.008
INS180’	−.246*	.030
Course	.312**	.001
SBP	.241**	.005
DBP	−.053	.539

** Significant correlation at 0.01 level; * significantly correlated at the 0.05 level

Spearman correlation analysis (Table 4) showed that: a positive correlation (the correlation coefficient was positive) was found between the accumulation of skin AGEs and the risk scores of the four major complications of diabetes (CVD risk score, DPN risk score, DR risk score, and DN risk score), renal function indicators (Cr, CysC, Hcy, ACR, MAU, α-MG, Tf, and U-Ig G), inflammatory indicators (NEUT, NLR, and CRP), and TCSS score. However, a negative correlation was found with LY, HOMAIR, FINS and eGFR (negative correlation coefficient).

**Table 4:** Spearman correlation analysis of AGEs and other indicators

Indicators	r_**s**_	***P***
CVD risk score	.831**	.000
DPN risk score	.756**	.000
DR risk score	.813**	.000
DN risk score	.818**	.000
TG	−.040	.346
HDL-C	.039	.366
Cr	.395**	.000
CysC	.381**	.000
Hcy	.232**	.000
ACR	.179**	.003
eGFR	−.434**	.000
MAU	.248**	.000
α-MG	.354**	.000
Tf	.303**	.000
Ig	.257**	.000
NEUT	.221**	.000
LY	−.117**	.006
NLR	.248**	.000
CRP	.143**	.001
HOMAIR	−.313**	.006
PFG	.037	.398
FINS	−.241*	.035
C-Peptide	−.022	.727
2hC-Peptide	−.086	.167
HbA1c	.003	.945
TCSS score	.454**	.000

** Significant correlation at .01 level; * significantly correlated at the .05 level

#### Comparison of AGEs among groups in DPN grades

One-way ANOVA (Table 5): Within the classification of VPT, CPT and TCSS, significant differences in AGEs were found between groups (all *P* < 0.05). The linear and non-weighted significance of VPT and TCSS groups is all < 0.05, indicating a linear relationship between the differences.

**Table 5:** Comparison of AGEs cumulants among different DPN grades

Group	Classification	N	mean	Standard deviation	F	***P***
VPT	< 15	229	81.05	14.067	26.335	0.000*
	15–25	207	85.38	16.099		
	> 25	124	93.65	17.067		
CPT	Vormal	327	83.36	13.467	8.717	0.000*
	Less	196	89.28	18.624		
	Allergy	37	83.35	20.996		
TCSS	Normal	78	77.86	13.886	35.426	0.000*
	Mild	339	82.64	13.515		
	Moderate	136	95.95	18.087		
	Severe	7	99.54	20.24		

*The significance level is 0.05

**Fig. 3:**
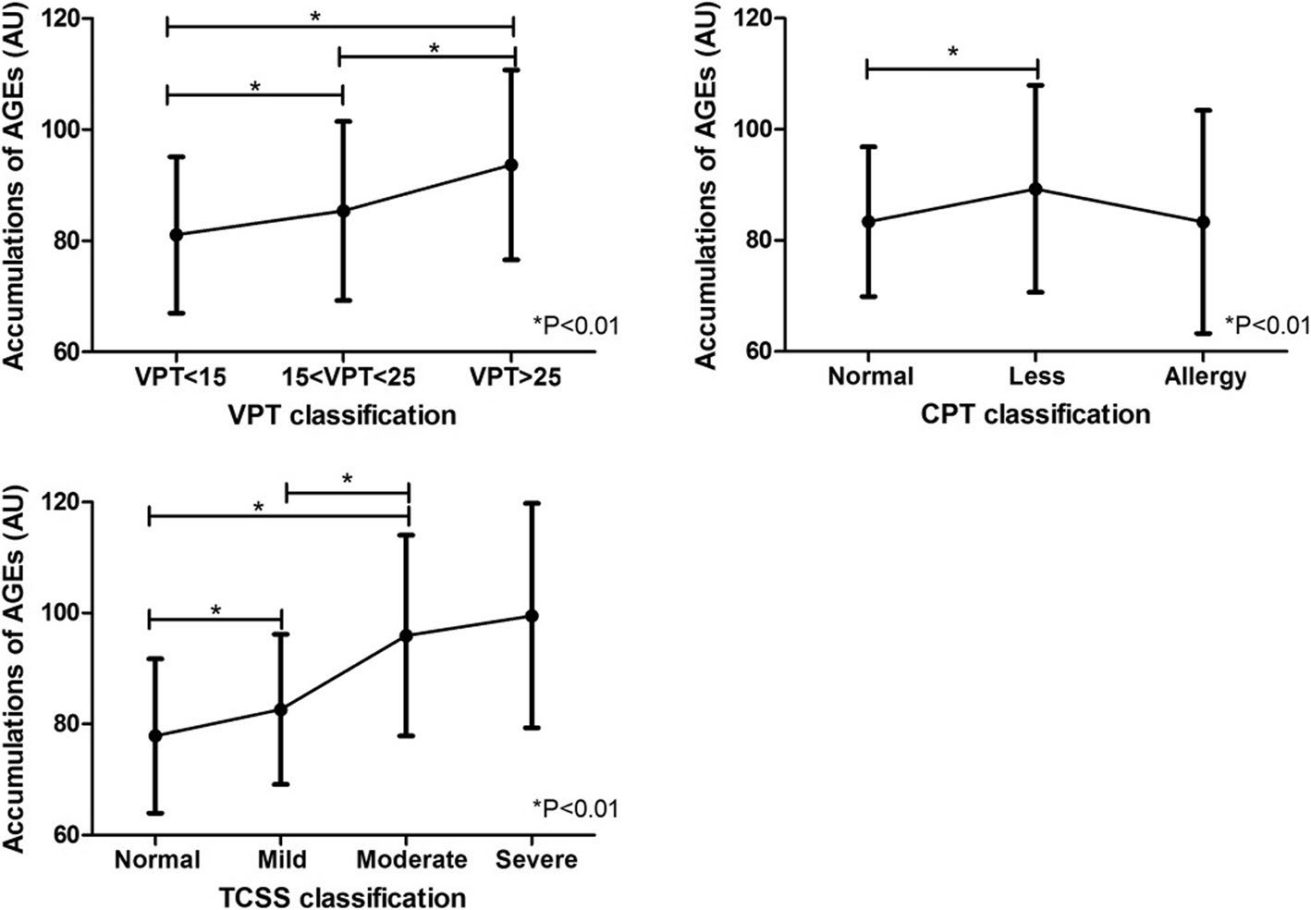
Correlation between AGEs and detection method of DPN

Multiple comparisons are shown as follows (Fig. 3): With the increase of VPT, AGEs accumulations increased gradually. Significant differences were found between VPT < 15, VPT 15–25, and VPT > 25 and between VPT 15–25 and VPT > 25 (*P* < 0.01). The cumulative amount of AGEs in the CPT normal group was significantly lower than that in the less group. A significant difference in AGEs was found between the CPT normal and the less groups (*P* < 0.01). With the increase of TCSS grading, the cumulative amount of AGEs increases, Significant differences were found between the TCSS normal group and those in the mild and moderate groups (*P* < 0.01 or *P* < 0.05). The difference between the mild and moderate group was found significant (*P* < 0.01).

#### ROC curve

**Fig. 4:**
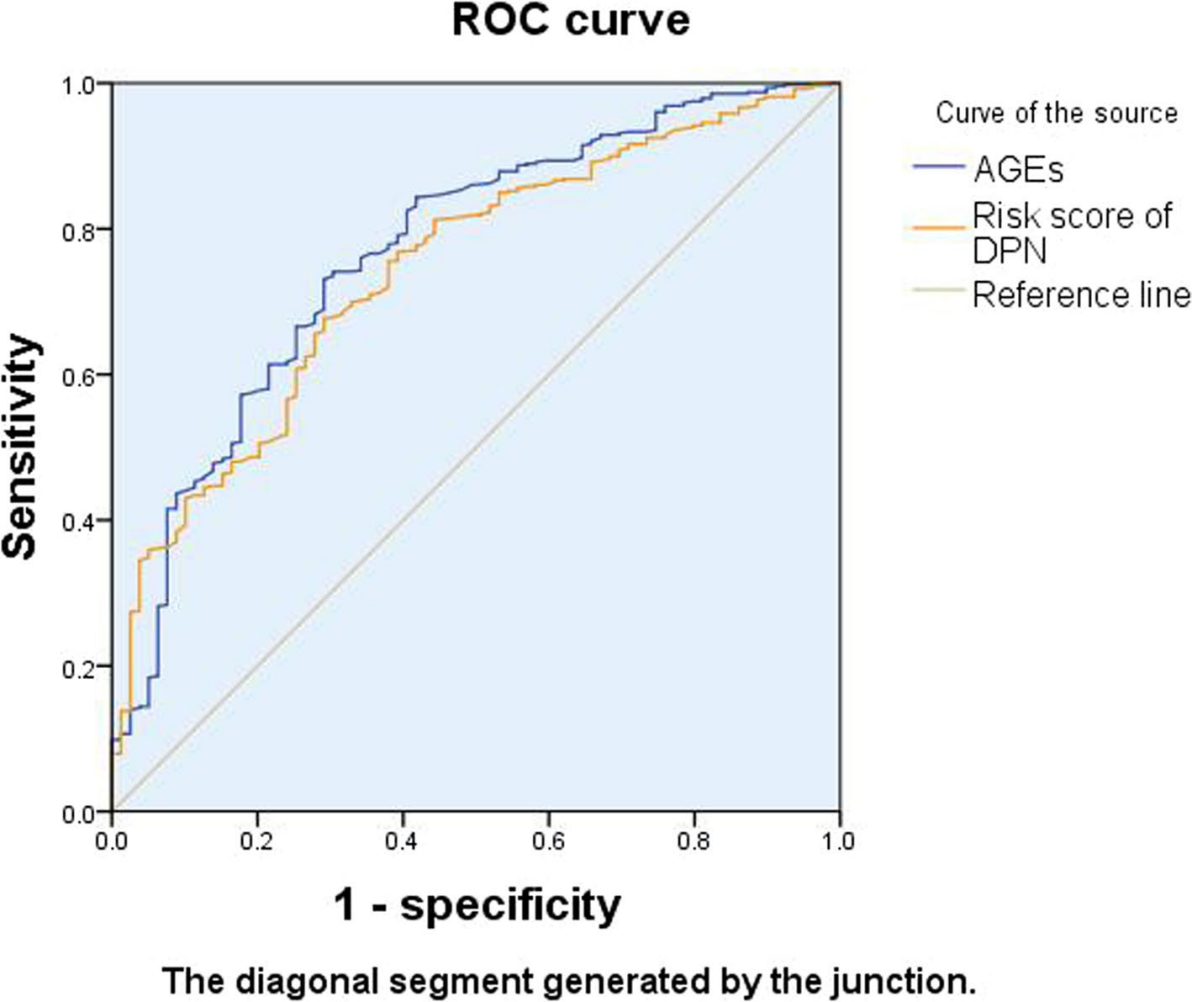
ROC curve of AGEs cumulant and neuropathy risk score

Figure 4 shows that the area under the curve of AGEs cumulant and neuropathy risk value is over 0.5, indicating that both AGEs cumulant and neuropathy risk value have certain accuracy in diagnosing DPN.

Significance *P* = 0.000 < 0.05, the area under the curve of AGEs cumulant and neuropathy risk scores was 0.769 and 0.743, respectively. The confidence intervals were (71.2–82.6%) and (68.8–79.9%), respectively, indicating that both of them were significant in the diagnosis of DPN, and the accumulative value of AGEs was more valuable in the diagnosis of DPN than neuropathy risk scores (Table 6).

**Table 6:** Area under the curve

Test variable	AUC	SE ^**a**^	Asymptotic sig.^**b**^	95% CI	
				Lower bound	Upper bound
AGEs	.769	.029	.000	.712	.826
Neuropathy risk score	.743	.028	.000	.688	.799

Test variable: AGE, neuropathy scores had at least one knot between positive and negative actual status groups. Statistics can be biased. ^a^under nonparametric assumptions; ^b^null hypothesis: real area = 0.5

The maximum Youden’s index of AGEs cumulant was 0.440, and the corresponding AGEs cumulant value was 77.65, which could be used as the critical value of DPN diagnosis. The corresponding sensitivity and specificity were 0.731 and 0.709, respectively. Similarly, the maximum Youden’s index of neuropathy risk value was 0.385, and the corresponding neuropathy risk value was 66.25, which could be used as the critical value of DPN diagnosis. The corresponding sensitivity and the specificity were 0.676 and 0.709, respectively (Table 7).

**Table 7:** Curvilinear coordinates

Test variable	Positive if greater than or equal to ^a^	Sensitivity	1-Specificity	Specificity	Youden index
AGEs cumulants	77.65	.731	.291	0.709	0.440
Neuropathy risk score	66.2500	.676	.291	0.709	0.385

Test variable: AGE, neuropathy scores had at least one knot between positive and negative actual status groups. ^a^The minimum boundary value is the minimum observation and test value minus 1, and the maximum boundary value is the maximum observation and test value plus 1. All other boundary values are the average of two adjacent observational test values

## Discussion

The first experimental report in 2004 [15] proposed that skin fluorescence as a noninvasive evaluation method was associated with AGEs values obtained from skin biopsies of patients with diabetes and healthy controls. This method was also recognized as the skin fluorescence ability to reflect AGE levels and was the basis of subsequent studies. However, until now, studies on peripheral neuropathy and AGEs of T2DM are few. Karabouta Z et al. [16] had confirmed that minors with T2DM were different from those with T1DM. Patients with T2DM could develop peripheral neuropathy soon after diagnosis. Thus, a feasible and straightforward method (indicator) is required to effectively screen such populations and can be used as an indicator of treatment or follow-up.

Ye Chengsong et al. [17] confirmed that compared with the normal group, the skin AGEs and serum AGEs detected by fluorescence spectrometry were significantly higher in diabetic patients, indicating that the skin fluorescence AGEs test had the following advantages: noninvasive, sensitive, and high specificity. The Chinese Academy of Sciences’ successful use of this spectral technique was achieved by using full-wavelength reflectance to correct the interference caused by differences in subjects’ skin color. The result was superior to the international use of ultraviolet reflectance only. This had laid a solid foundation for the noninvasive detection and promotion of glycation products. DM-Scan, developed and designed, was noninvasive, fast, easy to operate, repeatable, and accurate. The technology and equipment were suitable for rapid risk screening and follow-up of diabetes and complications in developing countries with large populations.

It was found in this study (Tables 1 and 2, Fig. 2) that with the increase of the severity of DPN, the accumulations of AGEs and the risk scores of the four major complications showed an increasing trend, indicating that all of them had certain reference significance for grading the severity of DPN lesions. It could also be inferred that the four major complications of diabetes have the same pathogenesis. In addition, no significant difference was found between grades 2 and 3 of AGEs in this study, which might be related to the inconsistency of sample size, and might also indicate that its specificity in grade 2 and 3 was weak, so further studies were needed in the future.

Homocysteine (Hcy) may stimulate the production of oxygen free radicals through a large amount of accumulation in the body, which may lead to the emergence and aggravation of oxidative stress, reduce the NO production, and damage the vascular endothelium. In turn, it creates conditions for the accumulation of lipid plaques and the formation of thrombosis, and accelerates the ischemia and hypoxia of microvessels and peripheral nerve tissues. At the same time, it can directly kill neurons, damage the nucleus, mitochondria and others. Cystatin C is a link in the process of Hcy increase. It can also induce inflammation and affect arterial endothelium and function, thereby indirectly affecting nerve tissue [18]. Our study showed a significant difference between DPN and Cys C in varying degrees was observed. However, they need more resounding basic experimental demonstration in generation and pathogenic mechanisms.

DPN has been proven to be a chronic inflammatory state, and low levels of inflammation played a unique role in T2DM and its complications, and inflammation was correlated with vascular endothelial dysfunction and blood cell coagulation. Traditional inflammatory indicators including white blood cell count could predict the pathological condition of chronic inflammation to a large extent. CRP could increase vascular endothelial factor, leading to the thickening of the basilar membrane of small vessels, and blood circulation disorder. Moreover, the immune function of CRP promoted the increase of white blood cells, which stimulated the activation of complement at the same time, and finally led to cell apoptosis [19]. Lymphocytes were often used as primers as a response indicator of systemic inflammation. The NLR was a novel marker reflecting a variety of chronic inflammatory diseases, which could reflect congenital and adaptive immune responses. Some studies suggested that NLR had a specific effect on DPN for studying inflammatory factors [20]. Previous studies had shown that if NLR levels in patients with type 2 diabetes increase, they were more likely to develop DPN [21, 22]. The results of our study were consistent with the above research. Our study showed that NLR, CRP and ly were different between patients with neuropathy and non-neuropathy, but there was no significant difference between patients with neuropathy of different severity. In addition, we could speculate whether the content of AGEs can be reduced by anti-inflammatory treatment.

Our study found that except eGFR decreased gradually with the aggravation of the disease, other renal function indexes (Cr, ACR, MAU,α-MG, Tf, IgG) increased roughly with the deepening of DPN severity. It was confirmed that DPN might have the same pathogenesis as diabetic small vessel disease. This was not clearly pointed out in previous studies. However, whether the above renal function indicators can be used as a reference for DPN diagnosis needs to be expanded and further studied.

The primary function of MAPK was to ensure the energy stability of tissue cells, which could be activated and promoted when energy is insufficient. Increased intracellular Ca^2+^ could increase AMPK activity and enhance autophagy function [23]. Related literature has also shown that small-fiber neuropathy is prone to G856D mutation. Nav1.7 ion channel was excessively opened, and the axis showed time-dependent degeneration, which was associated with increased intracellular calcium ion content and decreased ATP level [24]. However, our study found no significant statistical difference between the calcium ion concentration and DPN lesion grading, but there was a tendency to decrease step by step.

C-peptide in T2DM has pro-inflammatory and pro-sclerosing effects. Additionally, in recent years, C-peptide has been proven to cause regeneration of small fibers and reduce the necrosis of nerves and vascular endothelium, which might be related to the enhancement of Na^+^-K^+^-ATPase activity and the promotion of NO release [25]. A cross-sectional study has shown that IR often plays a special role in the course of DPN in patients with metabolic syndrome [26]. A Korean study [27] concluded that IR was independently correlated with neuropathy in patients with T2DM. In this study, no significant differences were found between the groups in C-peptide and HOMA-IR, which might be because most of the patients in this study were in the late stage of T2DM, the pancreatic islet function was generally poor, and sufficient sample size was not included.

Studies have found that low-density lipoprotein in diabetic patients is easily affected by glycation, forming AGEs-LDL, which slows down the metabolism of LDL-c, reduces the clearance, and makes it more difficult to reduce blood lipid. After phagocytosis in the body, it was easy to form complexes that gather on the vascular endothelium and cross-link with each other, and might cause severe inflammation through NF-KB factors, eventually leading to vascular lesion and neuropathy [28]. In our study, no significant correlation was found between LDL-c, TC, and other lipid indicators and DPN, which might be related to the long-term adherence to diet education of diabetic patients in our hospital department and the active clinical intervention of lipids.

Our research showed that age, course, ACR, Cr, MAU,α-MG, Tf, Ig,CRP and NLR were directly proportional to the severity of diabetic neuropathy and AGE. In addition, LY, eGFR and insulin 1–2 h after the meal were negatively correlated with the severity of diabetic neuropathy and AGEs. It indicated that interoperability might exist between these indicators and AGEs. We speculated that AGEs might cause severe diabetic neuropathy through a mechanism similar to those mentioned above. Thus, combining AGEs with these indicators was speculated to improve the detection rate of DPN and better evaluate the severity of DPN.

As can be seen from Table 5 and the broken line statistics (Fig. 3), with the increase of VPT, the accumulations of AGEs gradually increased. The cumulative amount of AGEs in the CPT normal group was smaller than that in the weakened group. TCSS showed that the cumulative amount of AGEs increased with the degree (however, in this study, TCSS judged that the heavy group had little relationship with other groups, which might be related to the small sample size of this group). In the four DPN grading methods mentioned above, the accumulations of AGEs gradually increased with the increasing degree of DPN. The vibration perception threshold (VPT),the current perception threshold (CPT) and the Toronto Clinical Scoring System (TCSS) can detect DPN presence and severity. However, those diagnosis methods of DPN are easily affected by subjective feelings of patients while AGEs is an objective, economic indicator that can be more effective in predicting DPN. Furthermore, these methods ignore the screening of asymptomatic neuropathy. Thus, the accumulation of skin AGEs was inferred to evaluate and predict the grading of DPN and its severity.

According to the ROC curve, the cumulative value of AGEs and neuropathy risk scores had high sensitivity and specificity, and the former was more prominent. Therefore, both the cumulative value of ages and the risk value of neuropathy could be used as predictors for DPN diagnosis, especially age. The skin AGEs detection system obtained both results for detection and evaluation, so AGE Reader is speculated to screen DPN better.

The noninvasive detection system of glycosylated products used in this study was provided by Hefei Institutes of Physical Sciences, Chinese Academy of Sciences, model DM-Scan (AGE Reader). AGE Reader, according to specific emission wavelength fluorescence intensity and the amount of which accumulated in the skin of the principle of positive correlation, in safe light triggers the human skin, through the whole wavelength reflectance correction subjects including skin color difference of interference (different from foreign countries, UV reflectivity is used for correction, and the correction effect is better, and more suitable for the yellow race). Based on the optical signal response subjects which accumulate in the body, and in combination with the built-in relevant model (with a variety of diabetes complications, oxidative stress, and aging), the risk assessment of diabetes and its complications, detection of long-term control of diabetes, and assessment of oxidative stress and human aging can be achieved.

## Conclusion

The cumulative amount of skin AGEs can be used as the diagnostic index and DPN predictor. With high sensitivity, high specificity, and economic and social value, AGE Reader can be used as a reliable detection tool and be popularized.

### Limitations and prospects

This study has several limitations. First, this study was mainly cross-sectional. Because of time constraints and other reasons, a longitudinal observation was not possible. Second, although the diagnostic criteria and exclusion criteria were limited for the included cases, it was still impossible to avoid the influence of food, cooking methods, related drugs and other factors on ages.

For the abovementioned limitations, the following methods can be adopted for improvement: First, dynamically track the changes of patients’ AGEs in treatment, and intensely discuss the mechanism between AGEs and diseases. Next, conduct a detailed investigation report and guidance of diet and treatment for DPN patients and analyze the roles of influential factors of AGEs in the occurrence and development of diseases.

## Data Availability

The datasets used and/or analysed during the current study are available from the corresponding author on reasonable request.
